# Association between chronic pain and acute coronary syndrome in the older population: a nationwide population-based cohort study

**DOI:** 10.1186/s12877-023-04368-1

**Published:** 2023-10-31

**Authors:** Yu-Chang Liu, Chung-Han Ho, Yi-Chen Chen, Chien-Chin Hsu, Hung-Jung Lin, Chia-Ti Wang, Chien-Cheng Huang

**Affiliations:** 1 Department of Emergency Medicine, Chi Mei Medical Center, Tainan, Taiwan; 2 Department of Environmental and Occupational Health, College of Medicine, National Cheng Kung University, Tainan, Taiwan; 3 Department of Medical Research, Chi Mei Medical Center, Tainan, Taiwan; 4 Department of Information Management, Southern Taiwan University of Science and Technology, Tainan, Taiwan; 5 School of Medicine, College of Medicine, National Sun Yat-sen University, Kaohsiung, Taiwan; 6 Department of Emergency Medicine, Taipei Medical University, Taipei, Taiwan; 7 Department of Emergency Medicine, Kaohsiung Medical University, Kaohsiung, Taiwan

**Keywords:** Acute coronary syndrome, Chronic pain, Older

## Abstract

**Background:**

Chronic pain (CP) may increase the risk of acute coronary syndrome (ACS); however, this issue in the older population remains unclear. Therefore, this study was conducted to clarify it.

**Methods:**

We used the Taiwan National Health Insurance Research Database to identify older patients with CP between 2001 and 2005 as the study cohort. Comparison cohort was the older patients without CP by matching age, sex, and index date at 1:1 ratio with the study cohort in the same period. We also included common underlying comorbidities in the analyses. The risk of ACS was compared between the two cohorts by following up until 2015.

**Results:**

A total of 17241 older patients with CP and 17241 older patients without CP were included in this study. In both cohorts, the mean age (± standard deviation) and female percentage were 73.5 (± 5.7) years and 55.4%, respectively. Spinal disorders (31.9%) and osteoarthritis (27.0%) were the most common causes of CP. Older patients with CP had an increased risk for ACS compared to those without CP after adjusting for all underlying comorbidities (adjusted sub-distribution hazard ratio [sHR] 1.18; 95% confidence interval: 1.07–1.30). The increasement of risk of ACS was more when the follow-up period was longer (adjusted sHR of < 3 years: 1.8 vs. <2 years: 1.75 vs. <1 year: 1.55).

**Conclusions:**

CP was associated with an increased risk of ACS in the older population, and the association was more prominent when the follow-up period was longer. Early detection and intervention for CP are suggested in this population.

## Background

Chronic pain (CP) is one of the major healthcare problems worldwide, which is defined as a persistent or recurrent pain lasting longer than 3 months by the International Association for the Study of Pain [1, 2]. Previous studies have reported that the prevalence rate of CP ranged from 8.7 to 64.4% [3] and is more prominent in the female population and older people [4]. In addition to the original cause of CP, CP may contribute to impaired functional status, disability, decreased quality of life, increased comorbidities, death, and increased social and economic burdens [5–8].

CP is a stress on the human body, which may increase the risk of the acute coronary syndrome (ACS) [9–11]. A previous study reported that CP increased the rate of ACS events in non-older adults (< 65 years) (adjusted hazard ratio [AHR] 1.2; 95% confidence interval [CI] 1.0 − 1.4) [9]. Older people have a higher risk of CP than the younger population because of multiple comorbidities, including osteoarthritis, spinal disorders, peripheral vascular diseases, osteoporosis, and malignancy [12, 13]. The risk of ACS in older people with CP may be different from that in the younger population. However, the association between CP and ACS in this population remains unclear. Therefore, this study was conducted to clarify it and fill the data gap.

## Methods

### Data source

This nationwide population-based cohort study was conducted using Taiwan’s National Health Insurance Research Database (NHIRD) [14], which is one of the biggest health databases worldwide. The Taiwan National Health Insurance (NHI) program was launched in 1995, providing coverage to almost the entire Taiwanese population. Hence, the NHIRD can be regarded as representative of the general population in Taiwan [14]. The NHIRD contains complete data of all medical claims covered by the NHI, including outpatient, inpatient, and emergency department services. Many studies based on the NHIRD have provided useful information for clinical care [15]. In addition, the NHIRD provides real-world big data for researchers to investigate many important issues that are difficult to conduct by randomized controlled trials [15].

### Study Design, setting, and participants

**Fig. 1:**
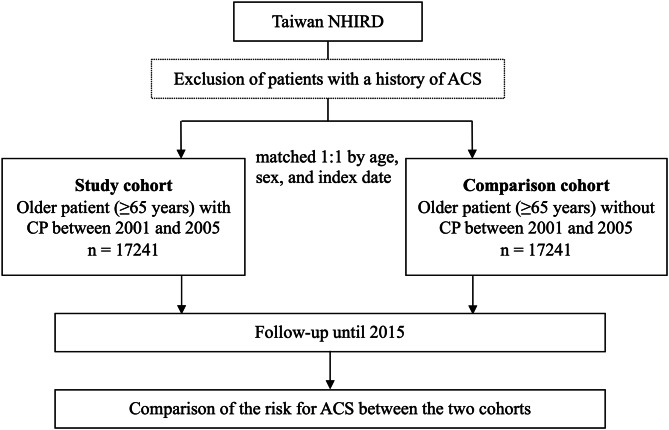
Flowchart of this study ACS, acute coronary syndrome; CP, chronic pain; NHIRD, National Health Research Database

After exclusion of patients with a history of ACS, we identified all older patients (≥ 65 years) with CP between 2001 and 2005 as the study cohort (Fig. 1). The diagnosis of CP was defined as the patient who has used analgesics, including non-steroidal anti-inflammatory drugs (excluding aspirin), acetaminophen, and opioids for more than 3 months [8, 9, 12, 16]. The index date was defined as the date when patients fit the diagnostic criteria for CP, specifically the date when they had used analgesics for a minimum of three months. We identified older patients without CP as the comparison cohort by matching age, sex, and index date with the study cohort at a ratio of 1:1 through sampling without replacement [17]. In consideration of examining more variables, we made the decision to minimize the matched variables, focusing on age, sex, and index date as the key matching criteria. For the subgroup analyses, we employed adjustments for all baseline characteristics presented in Table 1 in the multivariable model to minimize any potential confounding effects. In clinical decision making, it is essential to have information about the difference in ACS risk between older patients with and without CP. This knowledge helps clinicians determine whether to pay more attention to identifying CP and prescribing more intensive pain treatment. Therefore, this study was designed to compare the ACS risk between older patients with CP and those without CP, as it has more practical implications for decision making than knowing the absolute risk.

**Table 1:** Comparison of demographic characteristics between older patients with and without CP

Variables	Older patients with CP n = 17241	Older patients without CP n = 17241	*Standardized difference*
Age (mean ± SD)	73.5 ± 5.7	73.5 ± 5.7	0.00
Age subgroups (%)			
65–74 years	10099 (58.6)	10099 (58.6)	0.00
75–84 years	6357 (36.9)	6357 (36.9)	0.00
≥85 years	785 (4.5)	785 (4.5)	0.00
Sex (%)			
Male	7691 (44.6)	7691 (44.6)	0.00
Female	9550 (55.4)	9550 (55.4)	0.00
Underlying comorbidity (%)			
Hypertension	9634 (55.9)	6521 (37.8)	0.37
Diabetes	3768 (21.9)	2617 (15.2)	0.17
Hyperlipidemia	2182 (12.7)	1475 (8.6)	0.13
Coronary artery disease	3412 (19.8)	2248 (13.0)	0.18
Malignancy	1013 (5.9)	634 (3.7)	0.10
Stroke	2733 (15.9)	1485 (8.6)	0.22
Dementia	407 (2.4)	261 (1.5)	0.06
Congestive heart failure	2406 (14.0)	1535 (8.9)	0.16
Chronic obstructive pulmonary disease	3313 (19.2)	1882 (10.9)	0.23
Liver diseases	1633 (9.5)	1056 (6.1)	0.13
Renal diseases	1440 (8.4)	891 (5.2)	0.13
Connective tissue disease	408 (2.4)	180 (1.0)	0.10
Mental disorder	7452 (43.2)	4931 (28.6)	0.31

Data are presented as number (%) or mean ± SD

Abbreviations: CP, chronic pain; SD, standard deviation

### Definitions of variables

We categorized older patients into three distinct subgroups based on age: 65–74 years (youngest-old), 75–84 years (middle-old), and ≥ 85 years (oldest-old). This classification aligns with the categorization commonly used in the field of geriatric medicine [12, 18, 19]. The underlying comorbidities were identified according to the International Classification of Diseases, Ninth Revision, Clinical Modification (ICD-9-CM) codes, as the follows: hypertension (ICD-9-CM: 401–405), diabetes (ICD-9-CM: 250), hyperlipidemia (ICD-9-CM: 272), coronary artery disease (ICD-9-CM: 410–414), malignancy (ICD-9-CM: 140–208), stroke (ICD-9-CM: 436–438), dementia (ICD-9-CM: 290), congestive heart failure (ICD-9-CM: 428), chronic obstructive pulmonary disease (ICD-9-CM: 496), liver disease (ICD-9-CM: 570–576), renal disease (ICD-9-CM: 580–593), connective tissue disease (ICD-9-CM: 710), and mental disorder (ICD-9-CM: 290–302 and 306–319). The causes of CP were spinal disorders (ICD-9-CM: 756.11, 756.12, 720–725, and 737.1–737.4), osteoarthritis (ICD-9-CM: 715), peripheral vascular diseases (ICD-9-CM: 443.8–444.9), gout (ICD-9-CM: 274), osteoporosis (ICD-9-CM: 733.0), headache (ICD-9-CM: 307.81, 784.0, and 346), malignancy (ICD-9-CM: 140–208), diabetic neuropathy (ICD-9-CM: 250.6 and 357.2), rheumatoid arthritis (ICD-9-CM: 714), and pressure ulcer (ICD-9-CM: 707). The underlying comorbidities and causes of CP were defined as diseases with at least one hospitalization or three outpatient visits within the year prior to the index date.

### Outcome measures

The primary outcome measure was defined as ACS development during the follow-up period. ACS was identified using the ICD-9-CM of 410 or 411 with at least one hospitalization [12]. All patients were followed from the index date until the development of the ACS, death, or until December 31, 2015 (last date in the database).

### Ethical statements

The study was conducted in accordance with the World Medical Association Declaration of Helsinki. This study was approved by the Institutional Review Board of Chi Mei Medical Center, Tainan, Taiwan (Applicant’s No: 11105-E01). Because NHIRD data have been de-identified, the need for written informed consent was waived. The waiver does not affect the rights and welfare of the participants.

### Statistical analyses

We used the standardized difference to compare baseline characteristics between the two cohorts [20, 21]. An absolute standardized difference of > 0.10 was considered to indicate imbalance [21]. Based on the non-parametric statistic of Kaplan-Meier methods, we estimated the probabilities of ACS during the follow-up period and performed log-rank tests to compare cumulative incidence curves between the two cohorts. To account for survival bias, we used the Fine and Gray competing risk models to calculate the sub-distribution hazard ratios (sHRs) and corresponding 95% confidence intervals (CIs) of the risk of ACS between the cohorts [22]. We adjusted for potential confounders, including sex, age subgroups, and underlying comorbidities as listed in Table 1 in the multivariable models [23]. Stratified analyses were conducted to investigate potential effect modification across different age subgroups, sex, underlying comorbidities, and follow-up periods. Statistical analyses were performed using SAS 9.4 for Windows (SAS Institute, Inc., Cary, NC, USA). A two-tailed *p*-value < 0.05 was considered significant.

## Results

### Patient characteristics

A total of 17241 older patients with CP and the same number of older patients without CP were identified in this study (Table 1). Following matching, no significant difference was found in the age and sex between the two cohorts. The mean age (± standard deviation) was 73.5 (± 5.7) years. The percentage of the subgroup aged 65–74 years was the highest (58.6%), followed by the subgroups aged 75–84 years (36.9%) and ≥ 85 years (4.6%). Among older patients with CP, there were more women than men (55.4% vs. 44.6%). Compared with older patients without CP, older patients with CP had a higher prevalence of underlying comorbidities, including hypertension, diabetes, hyperlipidemia, coronary artery disease, malignancy, stroke, congestive heart failure, chronic obstructive pulmonary disease, liver diseases, renal diseases, and mental disorders (standardized difference > 0.10).

### Causes of CP

The most common cause of CP was spinal disorders (31.9%), followed by osteoarthritis (27.0%), peripheral vascular diseases (17.2.%), gout (10.9%), osteoporosis (10.4%), headache (8.8%), and malignancy (5.9%) (Table 2). The subgroup aged ≥ 85 years had a higher prevalence of peripheral vascular diseases, osteoporosis, and pressure ulcer, but a lower prevalence of spinal disorders, headache, diabetic neuropathy, and rheumatoid arthritis than younger patients.

**Table 2:** Causes of CP in older patients with CP

Cause of CP*	Total n = 17241	65–74 years n = 10099	75–84 years n = 6357	≥ 85 years n = 785
Spinal disorders	5506 (31.9)	3236 (32.0)	2063 (32.5)	207 (26.4)
Osteoarthritis	4655 (27.0)	2690 (26.6)	1768 (27.8)	197 (25.1)
Peripheral vascular diseases	2961 (17.2)	1517 (15.0)	1274 (20.0)	170 (21.7)
Gout	1877 (10.9)	1130 (11.2)	675 (10.6)	72 (9.2)
Osteoporosis	1796 (10.4)	981 (9.7)	723 (11.4)	92 (11.7)
Headache	1522 (8.8)	963 (9.5)	522 (8.2)	37 (4.7)
Malignancy	1013 (5.9)	563 (5.6)	405 (6.4)	45 (5.7)
Diabetic neuropathy	635 (3.7)	413 (4.1)	211 (3.3)	11 (1.4)
Rheumatoid arthritis	416 (2.4)	299 (3.0)	103 (1.6)	14 (1.8)
Pressure ulcer	116 (0.7)	59 (0.6)	45 (0.7)	12 (1.5)

Data are presented as number (%)

*There might be multiple causes of chronic pain

Abbreviations: CP, chronic pain

### Comparison of subsequent ACS between the two cohorts

**Fig. 2:**
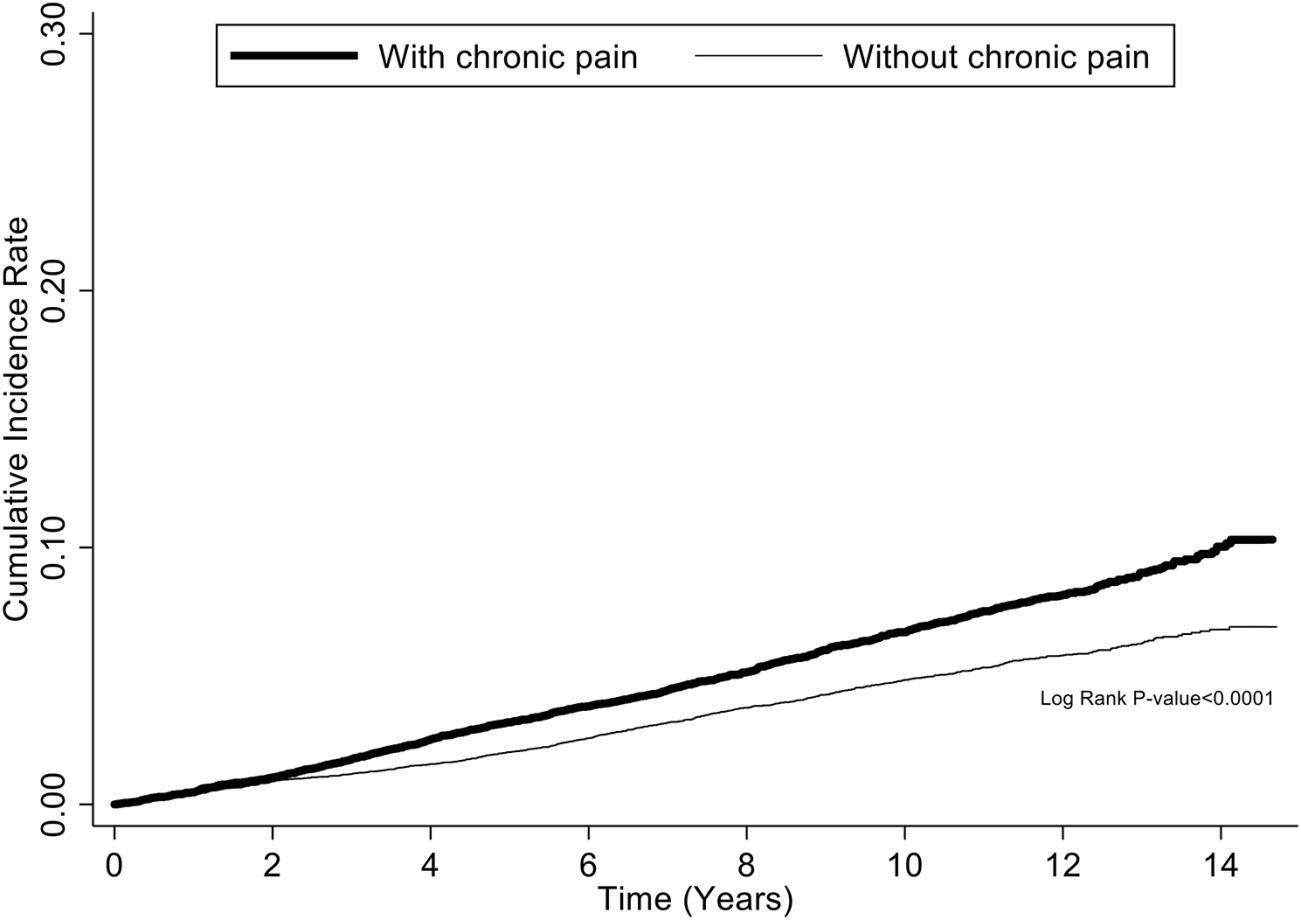
Kaplan–Meier curve and log-rank test for comparing the risk for acute coronary syndrome between older patients with and without chronic pain

The Kaplan–Meier method revealed that the cumulative incidence of ACS in older patients with CP was significantly higher than in those without CP during the follow-up period (log-rank test, p < 0.0001) (Fig. 2). The competing risk survival analysis revealed that older patients with CP had a higher rate of developing ACS than those without CP in both univariable (crude sHR 1.33; 95% CI 1.21–1.46) and multivariable models (adjusted sHR 1.18; 95% CI 1.07–1.30) (Table 3). Stratified analyses revealed that the elevated risk of ACS in older patients with CP was more prominent in the subgroup aged 65–74 years (adjusted sHR 1.21; 95% CI 1.06–1.39) compared to the subgroups aged 75–84 years (adjusted sHR 1.13; 95% CI 0.97–1.31) and ≥ 85 years (adjusted sHR 1.06; 95% CI 0.66 − 1.70). Furthermore, the increased rate of ACS events was statistically significant in both male (adjusted sHR 1.15; 95% CI 1.00 − 1.32) and female (adjusted sHR 1.21; 95% CI 1.05 − 1.39) patients. Additionally, patients with comorbidities of hypertension showed a heightened risk of ACS (adjusted sHR 1.18; 95% CI 1.04 − 1.34), as did those with chronic obstructive pulmonary disease (adjusted sHR 1.32; 95% CI 1.04 − 1.68). The risk of ACS increased with longer follow-up periods (adjusted sHR 1.80 for follow-up < 3 years vs. adjusted sHR 1.75 for follow-up < 2 years vs. adjusted sHR 1.55 for follow-up < 1 year).

**Table 3:** Comparison of the risk for ACS between older patients with and without CP using competing risk survival analysis

Variable	Older patients with CP		Older patients without CP		Crude sHR (95% CI)	Adjusted sHR (95% CI)*
	Mortality (%)	ACS (%)	Mortality (%)	ACS (%)		
Overall analysis	9061 (52.6)	1021 (5.9)	7782 (45.1)	776 (4.5)	1.33 (1.21 − 1.46)	1.18 (1.07 − 1.30)
Stratified analysis						
Age subgroups						
65–74 years	4169 (41.3)	558 (5.5)	3299 (32.7)	390 (3.9)	1.45 (1.27 − 1.65)	1.21 (1.06 − 1.39)
75–84 years	4215 (66.3)	426 (6.7)	3853 (60.6)	351 (5.5)	1.22 (1.06 − 1.41)	1.13 (0.97 − 1.31)
≥85 years	677 (86.2)	37 (4.7)	630 (80.3)	35 (4.5)	1.07 (0.67 − 1.69)	1.06 (0.66 − 1.70)
Sex						
Male	4478 (58.2)	517 (6.7)	4012 (52.2)	407 (5.3)	1.28 (1.13 − 1.46)	1.15 (1.00 − 1.32)
Female	4583 (48.0)	504 (5.3)	3770 (39.5)	369 (3.9)	1.38 (1.21 − 1.58)	1.21 (1.05 − 1.39)
Underlying comorbidity						
Hypertension	5087 (52.8)	677 (7.0)	3182 (48.8)	383 (5.9)	1.20 (1.06 − 1.36)	1.18 (1.04 − 1.34)
Diabetes	2253 (59.8)	317 (8.4)	1490 (56.9)	197 (7.5)	1.12 (0.94 − 1.34)	1.10 (0.92 − 1.31)
Hyperlipidemia	937 (42.9)	155 (7.1)	578 (39.2)	94 (6.4)	1.11 (0.86 − 1.44)	1.08 (0.83 − 1.40)
Coronary artery disease	1898 (55.6)	270 (7.9)	1157 (51.5)	156 (6.9)	1.14 (0.94 − 1.39)	1.13 (0.93 − 1.38)
Malignancy	741 (73.2)	34 (3.4)	392 (61.8)	20 (3.2)	1.07 (0.62 − 1.85)	1.04 (0.58 − 1.86)
Stroke	1737 (63.2)	192 (7.0)	966 (65.1)	102 (6.9)	1.02 (0.81 − 1.30)	1.01 (0.79 − 1.28)
Dementia	316 (77.6)	23 (5.7)	212 (81.2)	9 (3.5)	1.66 (0.77 − 3.59)	1.75 (0.79 − 3.89)
Congestive heart failure	1653 (68.7)	192 (8.0)	1063 (69.4)	120 (7.8)	1.02 (0.81 − 1.28)	1.03 (0.81 − 1.29)
Chronic obstructive pulmonary disease	2050 (61.9)	231 (7.0)	1150 (61.1)	95 (5.1)	1.39 (1.10 − 1.77)	1.32 (1.04 − 1.68)
Liver diseases	960 (58.8)	67 (4.1)	588 (55.7)	40 (3.8)	1.08 (0.83 − 1.60)	0.99 (0.66 − 1.47)
Renal diseases	944 (65.6)	117 (8.1)	581 (65.2)	70 (7.9)	1.04 (0.77 − 1.40)	0.99 (0.73 − 1.34)
Connective tissue disease	210 (51.5)	17 (4.2)	72 (40.0)	5 (2.8)	1.50 (0.56 − 4.08)	1.22 (0.46 − 3.22)
Mental disorder	4005 (53.7)	414 (5.6)	2432 (49.3)	237 (4.8)	1.16 (0.99 − 1.36)	1.09 (0.93 − 1.28)
Follow-up period						
< 1 year	61 (0.4)	19 (0.1)	55 (0.3)	11 (0.1)	1.73 (0.82 − 3.63)	1.55 (0.74 − 3.27)
< 2 years	140 (0.8)	30 (0.2)	132 (0.8)	17 (0.1)	1.77 (0.97 − 3.20)	1.75 (0.96 − 3.18)
< 3 years	233 (1.4)	46 (0.3)	171 (1.0)	23 (0.1)	2.00 (1.21 − 3.30)	1.80 (1.07 − 3.01)

* Adjusted for age subgroups, sex, and all underlying comorbidities, including hypertension, diabetes, hyperlipidemia, coronary artery disease, malignancy, stroke, dementia, congestive heart failure, chronic obstructive pulmonary disease, liver diseases, renal diseases, connective tissue disease, and mental disorder

Abbreviations: ACS, acute coronary syndrome; sHR, sub-distribution hazard ratio; CI, confidence interval; CP, chronic pain

## Discussion

This nationwide population-based cohort study reported that older patients with CP were at a higher risk of subsequent ACS than those without CP, similar to that in a previous study of the non-older population [9]. The increased risk of ACS was more prominent in the subgroup aged 65 − 74 years than in older age subgroups and in the group with a longer follow-up period than in those with a shorter follow-up period.

Our data fills the gap for older population about the association between CP and ACS in the literature. In addition, our study presented that the increased risk of ACS was more prominent in the group with a longer follow-up period, which indicates a possible dose–response relationship. A population-based cross-sectional study in New Zealand reported that cardiovascular diseases, including heart attack, heart failure, and other heart diseases, were independently associated with CP after adjusting for demographic factors and chronic physical conditions (odds ratio [OR] 1.6; 95% CI 1.3–1.9) [24]. Another large-scale population-based cohort study of extended families in Scotland also reported an increased co-occurrence of CP and cardiovascular disease (OR 4.19; 95% CI 3.64 − 4.82) [25]. A population-based cohort of adults aged < 65 years in Taiwan reported a significant association between CP and the risk of acute myocardial infarction (AHR 1.2; 95% CI 1.0 − 1.4). However, these aforementioned studies did not investigate the risk of ACS in older people. Our results were compatible with the findings of those studies and help complete a whole picture of CP and ACS in the total population.

One of the possible mechanisms for the increased risk of ACS is the effect of stress from CP. In response to stress, the hypothalamic–pituitary–adrenal (HPA) axis and the sympathetic nervous system release cortisol and catecholamines, which produce a negative effect on the vascular system by inducing high blood pressure, inflammation, and endothelial dysfunction [11, 26]. The dysregulation of the HPA axis may contribute to cardiovascular disorders, including ACS, left ventricular dysfunction, dysrhythmia, and atherosclerosis [10]. Furthermore, CP may cause a reduction of daily activities, sleep disturbance, depression, and cognitive decline [16, 27–29], which are also associated with increased cardiovascular diseases and death. However, the relationship between CP and cardiovascular diseases is complex and interdependent because of multiple physiologic, psychological, and even social factors [27]. Further studies are necessary to examine the exact underlying mechanisms.

In our study, we found that the increased risk for ACS was more prominent in the subgroup aged 65 − 74 years than in the subgroups aged 75 − 84 and ≥ 85 years. The possible explanation for the difference is that older people have more underlying comorbidities, such as hypertension, diabetes, and hyperlipidemia, responsible for cardiovascular diseases. Therefore, the combined effect decreases the role of CP played in the development of ACS.

This study has demonstrated that, while not all comorbidities significantly influence the association between CP and the risk of ACS, hypertension and chronic obstructive pulmonary disease do. These results suggest that individuals with hypertension or chronic obstructive pulmonary disease and CP may face a heightened risk of ACS compared to those with other comorbidities. The potential interaction between hypertension, chronic obstructive pulmonary disease, CP, and ACS has emerged as a novel finding, warranting further investigation into the underlying mechanisms.

The major strength of this study is attributed to its nationwide population-based design with a large sample size. In addition, this study, for the first time, clarified the association between CP and ACS in the older population. Despite the aforementioned strengths, it has some limitations. First, the claims-based database could not provide clinical information on some potential confounding factors, such as lifestyle, body mass index, smoking, and results of clinical tests. Nevertheless, we adjusted common underlying comorbidities related to these potential confounders to minimize the confounding effects. Second, there was no defined diagnostic code of CP in the NHIRD, so we used “analgesics usage for more than 3 months” as our criteria to identify CP. The diagnosis of CP may be underestimated, and the duration and severity of CP could not be further analyzed. Third, although we defined the use of analgesics as a criterion for CP, it is important to note that different types of analgesics may be associated with an increased risk of ACS. Previous epidemiological research conducted by our team on the older population with CP in Taiwan revealed that the most used analgesics were NSAIDs (89.1%), acetaminophen (77.5%), and opioids (10.2%) [12]. However, due to the frequent combination or alternation of analgesics, it becomes challenging to analyze the specific impact of a single type of analgesic on outcomes. As the primary aim of this study was to investigate the influence of CP on the risk of ACS in older patients, exploring the relationship between analgesics and ACS, including their specific types, may deviate from the focus of this study. Nevertheless, we acknowledge the importance of this aspect and plan to investigate the association between analgesic types and ACS in future research. Forth, the results may not be generalized to other nations because of the differences in race, culture, and medical insurance. Finally, we could not establish a causal relationship between CP and ACS because of the complex interactions and the observational study design. Further studies are warranted to delineate the issues mentioned above in the future.

## Conclusions

This nationwide population-based cohort study revealed a positive association between CP and ACS in the older population, especially in the longer follow-up period. The result fills the gap for older people in this issue. Possible mechanisms are as follows: CP causes stress, reduced daily activities, sleep disturbance, depression, and cognitive decline, which contributes to the increased risk of ACS. Therefore, we suggest a more aggressive control of CP in the older population to decrease the risk of subsequent ACS. Further studies, including recruiting more possible confounders, more precise diagnosis criteria of CP, and validation in other nations, are warranted in the future.

## Data Availability

Data are available from the NHIRD published by the Taiwan NHI Bureau. Due to legal restrictions imposed by the government of Taiwan in relation to the “Personal Information Protection Act,” data cannot be made publicly available. Requests for data can be sent as a formal proposal to the NHIRD (http://nhird.nhri.org.tw).

## Abbreviations

CP: chronic pain
ACS: acute coronary syndrome
AHR: adjusted hazard ratio
sHR: sub-distribution hazard ratio
CI: confidence interval
NHIRD: National Health Insurance Research Database
NHI: National Health Insurance
ICD-9-CM: International Classification of Diseases, Ninth Revision, Clinical Modification
OR: odds ratio
HPA: hypothalamic–pituitary–adrenal
